# Late Presentation of Paediatric Pink Pulseless Supracondylar Fracture of Humerus: A Case Report

**DOI:** 10.5704/MOJ.2003.019

**Published:** 2020-03

**Authors:** M Wong

**Affiliations:** Department of Orthopaedic Surgery, University of Malaya, Kuala Lumpur, Malaysia.

To the Editor,

I read with great interest the recent case reported by Kow *et al*^1^ regarding a six-year-old child with a supracondylar humerus fracture with a pink, pulseless hand presenting on the sixteenth day post trauma. The authors proceeded with an open reduction via anterior approach to explore the brachial artery. This is an unusual presentation and the child fortunately benefited from the procedure with improved cosmetic and functional outcome and no Volkman’s contracture complication. Being at sixteen days with radiographic evidence of callus in a very young child with a functional viable hand, it is interesting to deliberate on the need to explore the vessel?

There is little literature regarding guidelines for late presentation of supracondylar humerus fracture in children, so treatment remains controversial. The deformity may still be addressed later following union up till adolescence. This case is further compounded with the involvement of brachial artery injury which was partially transected and still in continuity but with good distal perfusion. This would have been a challenging dissection through callus and healing soft tissues.

Generally pulseless pink hands do not require exploration provided the distal limb remains well perfused after closed reduction and stabilization of supracondylar humerus fracture^2^. Exposed vessels run the risk of getting thrombosed, and there is the risk of compartment syndrome following vascular revascularization. Furthermore, exploration carries the risk of injury to collateral vessels which maintain a viable extremity^3^.
