# Supplementary material for: Late Presentation of Paediatric Pink Pulseless Supracondylar Fracture of Humerus: A Case Report
Source: Malays Orthop J. 2020 Mar;14(1):100–1. doi: 10.5704/MOJ.2003.019 (PMC7156170; doi:10.5704/MOJ.2003.019)
Supplement: Related texts

# Reply: Response to Letter to Editor

**DOI:** 10.5704/MOJ.1911.014

**Published:** 2019-09

**Authors:** Ren Yi Kow

**Affiliations:** 1 Department of Orthopaedic, Hospital Tengku Ampuan, Afzan (HTAA), Kuantan, Malaysia; 2 Department of Orthopaedic, International Islamic, University Malaysia (IIUM), Kuantan, Malaysia

Dear Sir,

Thank you for your interest in this case. To our best knowledge, this is the first case of late presentation of paediatric pink pulseless supracondylar fracture of the humerus in the literature. A literature review by Venkatadass K suggests close observation for patients with pink, pulseless humeral supracondylar fractures when the following criteria are met: 1) intact Doppler signal of the radial artery; 2) oxygen saturation >95% on pulse oximeter; and 3) preserved median nerve function^1^. The combination of absent radial artery Doppler signal and reduction of oxygen saturation indicates poor perfusion and early exploration is indicated^1^.

In our patient, there is loss of radial artery Doppler signal but a good blood oxygen saturation is preserved on pulse oxymetry. This is possibly due to collateral vessels supplying distal parts of the upper limb after a brachial artery injury. As the injury is 16-day-old, we deem that spontaneous recovery of the brachial injury is unlikely, as pulses are expected to return as late as 11 days after the injury^2^. As there is presence of callus at the fracture site, a trial of gentle traction is contraindicated in this case, as this might compromise collateral vessels. In this case, surgery is performed to reduce the fracture and for brachial artery exploration, bearing the possibility of arterial repair or grafting. By reducing the fracture, the patient will be benefitted from immediate pain relief as the sharp end of the proximal fracture fragment is impinging on the skin. Besides that, with early reduction of the fracture, we can prevent a poor functional outcome which necessitates an osteotomy later. On top of that, we can potentially prevent a dreaded complication such as Volkman’s contracture, possibly due to spasm of collateral vessels secondary to manipulation by non-medical personnel (traditional massage)^3^.

Nevertheless, open exploration and fixation may still impose a risk of damaging or causing spasm of collateral vessels, leading to ischemia of the hand. Facing this dilemma in which both options may lead to a devastating outcome, a good communication between the treating surgeon and the patient’s parents/guardian is of utmost importance. In this patient, we have performed an early open reduction and exploration after a family conference with her parents and they are aware of the possible sequelae of all options. Intra-operatively, the brachial artery is found to be partially transected with no active bleeding due to intraluminal thrombosis. After removal of blood clot, thrombolysis and brachial artery repair, the distal pulses return and are confirmed by Doppler examination at both radial and ulnar sites. Doppler monitoring of the arterial supply is performed daily prior to discharge.

## References

[1] Venkatadass K (2015). Pink pulseless hand - Evaluation and Decision making: Is there a Consensus?.. Int J Paediatr Orthop..

[2] Louahem D, Cottalorda J (2016). Acute ischemia and pink pulseless hand in 68 of 404 gartland type III supracondylar humeral fractures in children: Urgent management and therapeutic consensus.. Injury..

[3] Devnani AS (2005). Late Presentation of Supracondylar Fracture of the Humerus in Children.. Clin Orthop Relat Res..
